# Effect of Tongfu Xingshen capsule on the endogenous neural stem cells of experimental rats with intracerebral hemorrhage

**DOI:** 10.3892/mmr.2021.12263

**Published:** 2021-06-30

**Authors:** Zhizhong Cui, Shanshan Liu, Lingbo Hou, Yifan Sun, Haoxuan Chen, Hui Mao, Yuanqi Zhao, Lijun Qiao

**Affiliations:** 1 Second Clinical Medical College, Guangzhou University of Chinese Medicine, Guangzhou, Guangdong 510006, P.R. China; 2 First Clinical Medical College, Guangzhou University of Chinese Medicine, Guangzhou, Guangdong 510006, P.R. China; 3 Department of Hematology, Guangzhou Institute of Pediatrics, Guangzhou Women and Children's Medical Center, Guangzhou, Guangdong 510630, P.R. China; 4 Guangdong Provincial Hospital of Chinese Medicine, Guangzhou, Guangdong 510120, P.R. China

**Keywords:** Tongfu Xingshen capsule, hemorrhagic stroke, neural stem cells, brain protein, nutritional factor

## Abstract

Intracerebral hemorrhage (ICH) can stimulate neural regeneration, promoting tissue repair and recovery of nerve function. Tongfu Xingshen capsule (TXC) is a Chinese medicinal formula used to treat ICH and has been shown to protect brain tissue and improve nerve function in clinical studies. However, the effect of TXC on endogenous neural stem cells (NSCs) remains elusive. To explore the mechanisms underlying TXC action, a rat model of ICH was established. The effects of TXC on the proliferation and differentiation of NSCs were assessed in the subventricular zone (SVZ). TXC significantly improved nerve function defects, decreased brain water content and restored blood-brain barrier integrity. Additionally, BrdU labeling showed that both high and low doses of TXC significantly increased the proportion of actively cycling NSCs positive for Nestin and glial fibrillary acidic protein, but did not affect the proliferation rates of NeuN-positive neurons. Finally, TXC also upregulated the mRNA levels of brain-derived neurotrophic factor and its receptor, TrκB, in affected brain tissues. Taken together, TXC accelerated neural repair and functional recovery after brain injury by potentially enhancing the proliferation and differentiation of endogenous NSCs into astroglial cells in the SVZ area.

## Introduction

Intracerebral hemorrhage (ICH) is a type of stroke associated with higher morbidity and mortality rates (1). It is a significant cause of death and disability, with an incidence rate of 24.6 per 100,000 person-years and a fatality rate of 40% in 21 countries between 1980 and 2008 (2). ICH mainly results from the rupturing of small arterioles due to chronic hypertension (3). However, the pathophysiology of ICH is highly complex and involves multiple mechanisms. The poor outcome of ICH is attributed to both direct damages caused by hemorrhage and secondary injuries, such as brain edema, blood-brain barrier (BBB) disruption, inflammation and neuronal apoptosis (4). ICH also influences brain function, especially cognitive abilities, and may even cause cognitive decline or impairment (5). Indications for surgical treatment of ICH are restricted to a few clinically-relevant survival advantages (6). Although studies have recently focused on the pharmacological treatment of ICH, no effective regimen has been established so far (7).

Neural stem cells (NSCs) are characterized by self-renewal and multipotent differentiation (8). They can differentiate into neurons, astrocytes and oligodendrocytes, depending on the specific stimuli received (9). NSCs located in the lateral subventricular zone (SVZ) of the adult mammalian brain generate new neurons and glia throughout life (10). Additionally, NSCs play an important role in replenishing neural cells, neurotrophy and neuro-immunoregulation (11), promoting the recovery of motion and sensory and cognitive functions following any injury (12). Interestingly, ICH stimulates NSC proliferation and differentiation in the SVZ (13,14), and the nascent neurons then migrate to the damaged brain region to replace the dead neurons. Since neurogenesis plays a pivotal role in facilitating neurological recovery after stroke (15), NSC-based therapy has gained considerable attention for treating hemorrhagic stroke. A study demonstrated that NSC transplantation could promote the functional recovery of ICH rats (16). It has also been reported that endogenous NSCs are activated in the brain of experimental ICH rats and help neurons achieve self-repair (17). Neurotrophic factors, such as brain-derived neurotrophic factor (BDNF) and its receptor (TrκB), promote the proliferation and differentiation of endogenous NSCs (18) and may augment lesion repair.

Traditional Chinese medicine (TCM) has been used to improve health, prevent diseases, and treat serious illnesses for thousands of years in China and other Asian countries (19,20). The formulations consist of multiple herbs that target several disease components by following the TCM theory (21). Tongfu Xingshen capsules (TXC) are composed of senna leaf, giant knotweed rhizome, tabasheer, snake gourd seed and artificial bezoar to purge fu-organs to arouse the spirit, clear heat and resolve phlegm. It was first prescribed by Professor Liu of Guangdong Hospital of Traditional Chinese Medicine for treating hemorrhagic stroke as part of the ‘Tongfu-Xiedu-Xingnao-Kaiqiao’ treatment model. TXC can promote recovery after cerebral hemorrhage, improve capillary permeability of the hemorrhagic area, and alleviate cerebral edema. The absorption of hematoma can relieve pressure on the brain tissue, improve cerebral microcirculation and oxygen supply, and eventually restore neurological functions (22–25). In the present study, the mechanisms underlying the therapeutic effect of TXC was assessed in a rat model of ICH, with a specific focus on the neurological function score and the proliferation and differentiation of endogenous NSCs.

## Materials and methods

### 

#### Chemicals and reagents

Tongfu Xingshen capsules (TXC; 0.4 g/granule) were provided by the Pharmacy Department of Guangdong Hospital of Traditional Chinese Medicine. Evans Blue (EB), BrdU, formamide and sodium pentobarbital were purchased from Guangzhou Weijia Technology Co. Ltd. All chemicals and solvents were of an analytical grade.

#### Establishment of an ICH model and drug administration

Male SPF grade SD rats (age, 6 weeks) weighing between 200 and 250 g were purchased from the Institute of Experimental Animals, Chinese Academy of Medical Sciences (Beijing, China), and acclimatized in standard cages under controlled temperature (23±3°C) and a regular 12 h:12 h light-dark cycle for 7 days before the procedure. All experimental animal protocols were approved and performed following the Institutional Animal Care Guidelines (approval no. 2016040; Experimental Animal Ethics Committee, Guangdong Provincial Hospital of Chinese Medicine, Guangzhou, China).

The animals were divided into the sham-operated, untreated model, low dose TXC-treated, and high dose TXC-treated groups, and ICH was induced through autologous intracerebral blood infusion. In brief, the animals were all anesthetized using pentobarbital (50 mg/kg i.p.), and 50 µl blood was drawn from the tail artery. The rats were positioned in a stereotaxic frame (Stoelting Instruments), and a cranial burr hole (1 mm) was drilled near the right coronal suture 3.0 mm lateral to the midline. A microsyringe was stereotaxically inserted into the right basal ganglia (coordinates: 0.2 mm anterior, 6.0 mm ventral, and 3.0 mm lateral to the bregma), and autologous whole blood was injected at the rate of 10 µl/min (26–28). In the sham-operated control, the blood was also drawn from the tail artery before the needle was inserted without injecting any blood. The rats were intragastrically administered 1 ml/100 g normal saline (sham-operated and untreated controls), 1 g TXC/kg/day (high dose; 0.1 g/ml with normal saline), or 0.5 g TXC/kg/day (low dose; 0.05 g/ml with normal saline), as appropriate. For testing neurological function score, brain water content and vascular permeability test, 96 SD rats were divided as aforementioned (n=24 per group). Each group was further subdivided into the 1, 7, 14 and 28-day groups (n=6 per group). For immunofluorescence and reverse transcription-quantitative PCR (RT-qPCR), 72 animals were similarly divided into 7, 14 and 28-day subgroups in each treatment group.

#### BrdU labeling

BrdU (50 mg/kg) was intraperitoneally injected at a concentration of 5 mg/ml in 0.9% NaCl, once a day. Except for the 1-day group, the other groups received daily injections for 5 days after the model was established.

#### Tissue collection

From each rat, 6-µm thick serial coronal sections of the brain were cut to include the entire SVZ (80–90 slides per animal, and 3 sections per slide). Every tenth slide was stained using hematoxylin and eosin at room temperature following standard protocols. Briefly, brain sections were stained with hematoxylin for 5 min and differentiated with 1% hydrochloric acid alcohol for 5 sec at room temperature. Following immersion in ammonia-water for 10 sec, sections were dyed with eosin for 3 min, followed by mounting with neutral balsam. After sealing, the SVZ structures were observed at ×40 magnification under an Olympus BX61 light microscope, and adjacent sections with similar SVZ structures were used for double immunofluorescence staining.

#### Behavioral testing

Neurological tests were performed 24 h post-stroke and were scored according to the modified Bederson scale as follows: 0, no deficits; 1, flexed forepaw; 2, an inability to resist the lateral push; 3, circling; 4, agitated circling; and 5, unresponsive to stimulation/stupor (29). The neurobehavioral score of 1–5 was considered as a successful model.

#### Evan's Blue dye extravasation assay

Evan's Blue dye extravasation test was performed, as previously described (30). The harvested brains were quickly separated into the left anterior, right anterior, left posterior and right posterior segments, weighed and digested using 3 ml formamide in a 60°C water bath for 24 h. The homogenates were centrifuged at 10,000 × g at room temperature for 20 min, and the absorbance of the supernatant at 630 nm was measured using a spectrophotometer. The Evans blue content was calculated as mg/g against a standard curve.

#### Brain water content

Rats were decapitated under deep anesthesia (pentobarbital; 50 mg/kg i.p.), which was validated based on the fact that there was no pain reaction, no stimulation reaction, general muscle relaxation, and smooth breathing, and the brains were immediately removed. The posterior brains were weighed using an analytical microbalance to obtain the wet weight (WW). The samples were then dried at 75°C for 5 days, and the dry weight (DW) was determined. Brain water content was calculated as (WW-DW)/WW ×100%.

#### Double immunofluorescence

NeuN/BrdU and GFAP/BrdU double staining were performed on the 14-day and 28-day samples, while the 7-day samples were stained using Nestin and BrdU. In brief, the sections were incubated overnight with rat anti-BrdU (cat. no. ab6326) and mouse anti-Nestin (cat. no. ab6142), rabbit anti-NeuN (cat. no. ab177487) or rabbit anti-GFAP (all 1:200; cat. no. ab33922; all Abcam), as appropriate, at 4°C. After probing with the relevant secondary antibody (all 1:500; Alexa Fluor^®^488 donkey anti-rat, cat. no. A21208; anti-mouse, cat. no. A32744 or anti-rabbit IgG, cat. no. A32754; Thermo Fisher Scientific, Inc.) at room temperature for 1 h in the dark, the slides were washed and observed at ×400 magnification under a Nikon Ti2-E light microscope. ImageJ software (v2.1.4.7; National Institutes of Health) was used to measure the number of positive cells. The number of cells in the SVZ region of 3 non-overlapping fields were counted in each section, and the mean was calculated.

#### RNA preparation and RT-qPCR analysis

Total RNA was extracted from the brain tissues using the TRIzol reagent, following the manufacturer's instructions (Takara Biotechnology Co., Ltd.). The following primers were used to perform the RT-qPCR: Rat-BDNF forward primer, GATTAGGTGGCTTCATAGGAGAC; rat-BDNF reverse primer, CGAACAGAAACAGAGGAGAGATT; rat-TrκB forward primer, GATGTTCCAGCCACTGTGAACC; rat-TrκB reverse primer, TCCACCACCCTGTTGCTGTA. Rat-GADPH (forward, 5′-GATGTTCCAGCCACTGTGAACC-3′ and reverse, 5′-TCCACCACCCTGTTGCTGTA-3′) was used as the internal control. The purity of RNA samples was assessed via NanoDrop, and samples with ratios between 1.80 and 2.01 were used for cDNA synthesis using the GoScript™ Reverse Transcription system, according to the manufacturer's protocol (Promega Corporation). The qPCR reaction was performed in 20 µl with 5 µl template DNA and 500 nM primers. The PCR condition was as follows: Initial denaturation, 95°C for 10 min, 40 cycles of amplification (95°C for 10 sec and 60°C for 30 sec) and cooling period, 50°C for 5 sec. Each sample was analyzed in triplicate. iTaq™ Universal SYBR^®^ Green Supermix (Bio-Rad Laboratories, Inc.) was used according to the manufacturer's instruction. The PCR results were analyzed in a Real-Time PCR system (Bio-Rad Laboratories, Inc.; cat. no. CFX96). The specificity of the primers was tested by analyzing the dissolution curve. The mRNA of each target gene was homogenized, and the phase of mRNA of each target gene was calculated by Cq value for the relative expression level (2^−ΔΔCq^ method: ΔΔ Cq=(Cq _target gene_ -Cq _GAPDH_) _treatment group_- (Cq _target gene_ -Cq _GAPDH_) _control group_) (31).

#### Statistical analysis

The data were analyzed using SPSS 21 software (IBM Corp.) and are expressed as the mean ± SD. Differences were analyzed using one-way analysis of variance ANOVA with post hoc Bonferroni test. P<0.05 was considered to indicate a statistically significant difference.

## Results

### 

#### TXC alleviates neurological deficit and structural damage following ICH

**Figure 1.**
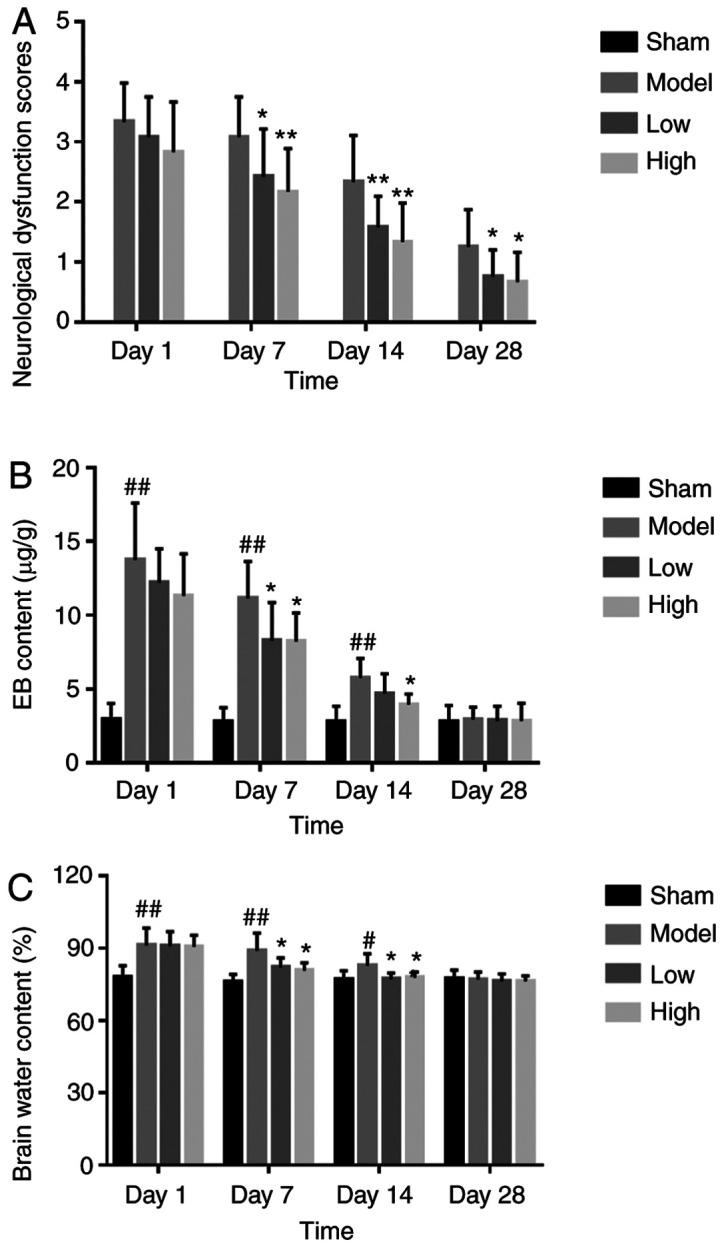
(A) Neurological scores in the groups indicated. (B) Evans Blue content of hemorrhagic brain tissue in the different groups. (C) Brain water content in the different groups. ^#^P<0.05 and ^##^P<0.01 vs. sham group; *P<0.05 and **P<0.01 vs. model group.

ICH resulted in left hemiplegia, severe hemiplegia in the left forelimb and clockwise rear-ended rotation within 7 days of induction, and the symptoms were observed over 14 and 28 days. Compared with the untreated model group, both low and high dosages of TXC improved gait symptoms and decreased hemiplegia (Fig. 1A). Furthermore, TCX significantly decreased the extravasation of EB from brain tissues after 7, 14 and 28 days of stroke, compared with the untreated model group (Fig. 1B). Finally, the brain water content in the ipsilateral hemicerebrum substantially increased in the model group, compared with the sham-operated animals, and was significantly decreased by low and high dose TXC treatment at all time points (Fig. 1C).

#### TXC promotes the proliferation and differentiation of endogenous NSCs in SVZ

**Figure 2.**
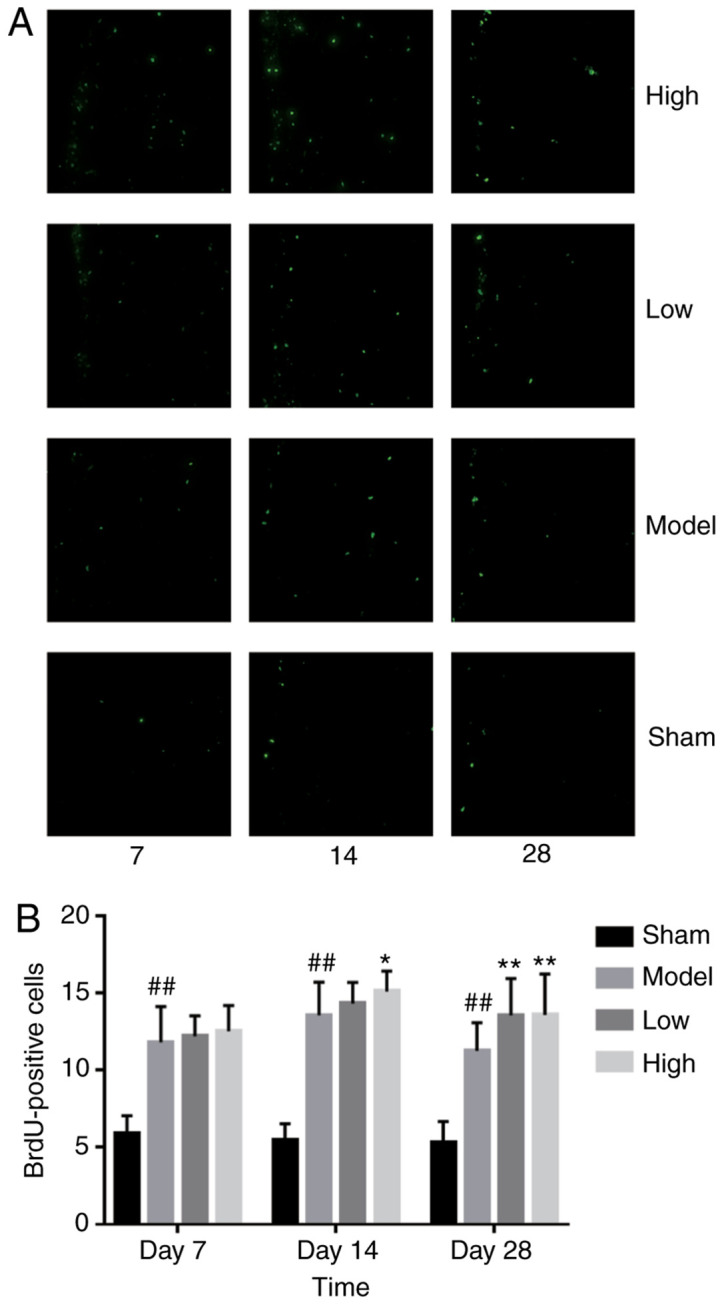
(A) Representative immunofluorescence images showing BrdU-positive cells (magnification, ×400; n=3). (B) The number of BrdU-positive cells in the SVZ of the different groups. ^##^P<0.01 vs. sham group; *P<0.05 and **P<0.01 vs. model group.

**Figure 3.**
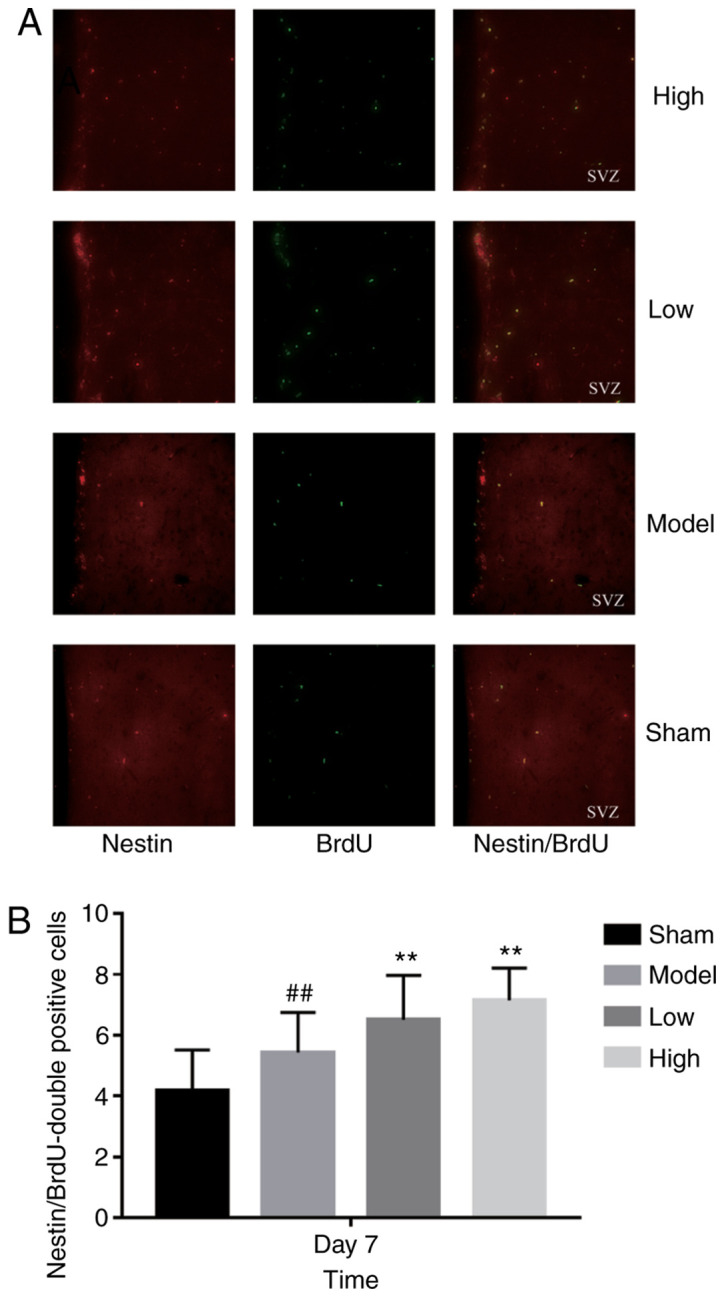
(A) Representative immunofluorescence images showing Nestin- and BrdU-positive cells (magnification, ×400; n=3). (B) The number of Nestin- and BrdU-positive cells in the subventricular zone of the different groups. ^##^P<0.01 vs. sham group; **P<0.01 vs. model group.

**Figure 4.**
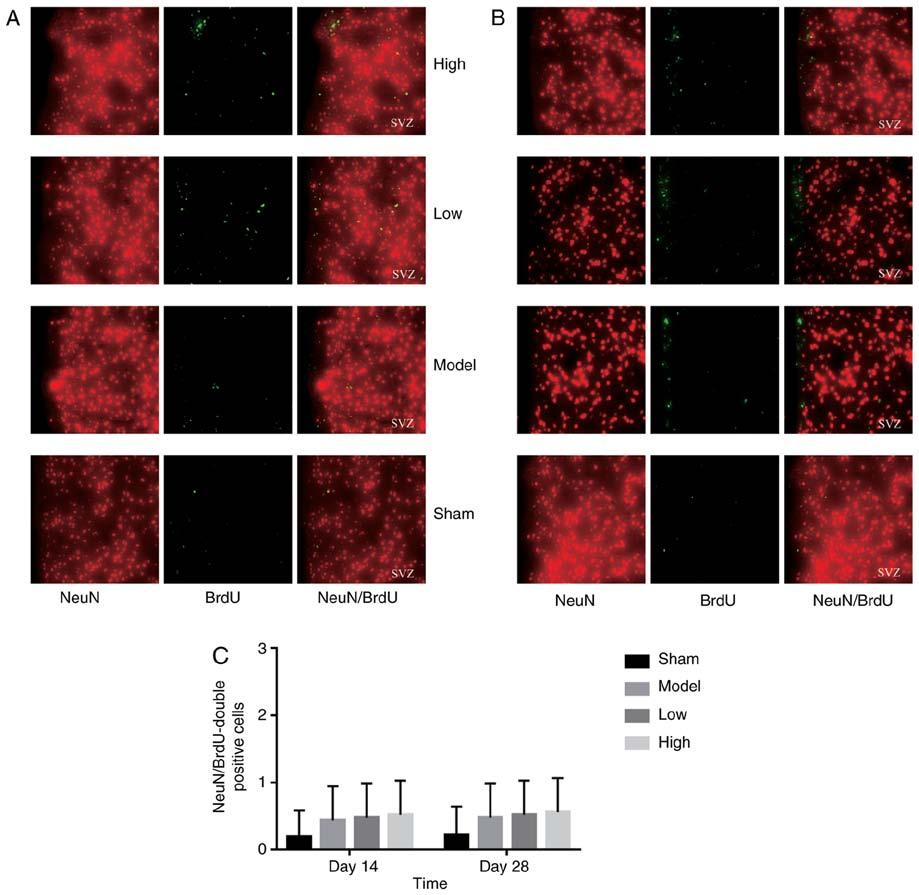
Representative immunofluorescence images showing NeuN- and BrdU-positive cells on (A) day 14 and (B) day 28 (magnification, ×400; n=3). (C) The number of NeuN- and BrdU-positive cells in the subventricular zone of the different groups.

**Figure 5.**
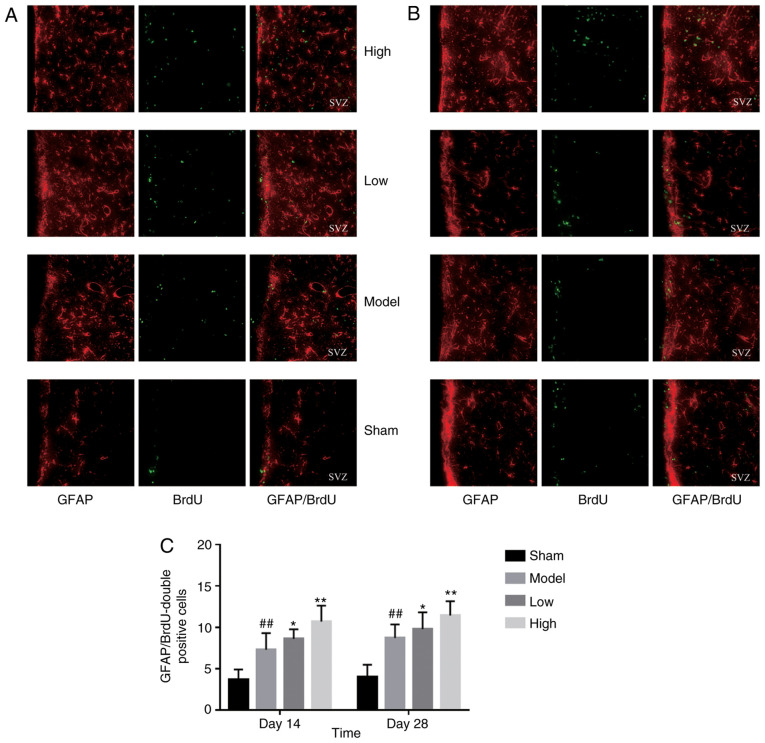
(A) Representative immunofluorescence images showing GFAP- and BrdU-positive cells on day 14 and (B) day 28 (magnification, ×400; n=3). (C) The number of GFAP- and BrdU-positive cells in the subventricular zone of the different groups. ^##^P<0.01 vs. sham group; *P<0.05 and **P<0.01 vs. model group. GFAP, glial fibrillary acidic protein.

ICH injury significantly increased the number of proliferating BrdU-positive cells in the SVZ region, increased by low or high dose TXC-treated (Fig. 2). As shown in Fig. 3, the total number of proliferating NSCs (Nestin- and BrdU-positive cells) was markedly increased after TXC treatment within 7 days post-stroke. However, the proportion of actively cycling neuron (NeuN- and BrdU-positive) cells was similar in both the untreated and treated groups, both at 14 and 28 days post-stroke (Fig. 4). Furthermore, TXC treatment also increased the number of proliferating glial cells (GFAP- and BrdU-positive) in the SVZ region after 14 and 28 days post-stroke (Fig. 5).

#### TXC upregulates BDNF and TrκB after ICH

**Figure 6.**
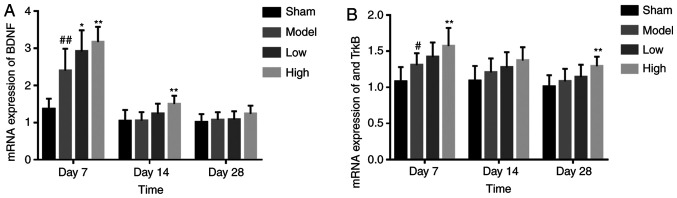
(A) BDNF mRNA expression in the SVZ of the different groups. (B) TrkB mRNA expression in the SVZ of the different groups. ^#^P<0.05 and ^##^P<0.01 vs. sham group; *P<0.05 and **P<0.01 vs. model group. SVZ, subventricular zone. BDNF, brain-derived neurotrophic factor.

The BDNF mRNA levels were significantly higher in the brain tissues of the TXC-treated animals compared with the untreated controls at 7 and 14 days post-stroke. The upregulation on day 14 was particularly notable in the high dose TXC group. However, there was no significant difference in BDNF expression between the untreated and treated groups after 28 days (Fig. 6A). TrκB mRNA was also upregulated in the model group compared with the sham-operated group after 7 days (P<0.05), and this effect was augmented further by high-dose TXC treatment on day 7 and day 28 (Fig. 6B).

## Discussion

It was found that EB extravasation in the brain was most significant on the first-day post-stroke and decreased along with time. Brain edema following ICH is mainly attributed to the disruption of the BBB (32). However, currently, drugs that can significantly improve BBB function after stroke have not yet been identified. TXC significantly inhibited EB extravasation, indicating that it can improve cerebral vascular permeability and alleviate brain edema of the affected rats in a dose-dependent manner during ICH.

A few BrdU-positive cells were detected in the ependymal epithelium of the SVZ region at different time points in the sham-operated group, which was consistent with the small number of resting endogenous NSCs previously reported in this region (13). In the present study, ICH significantly increased the number of proliferating Nestin-positive NSCs on the 7th day, which further enhanced the TXC treatment effect. Furthermore, significant glial cell proliferation was observed in the TXC-treated animals on the 14 and 28th days after TXC administration. TXC promoted NSC proliferation and differentiation into astroglial cells but not into neurons. However, the proliferation and activation of astrocytes were also beneficial for ICH. Astrocytes act as a ‘double-edged sword’ inactivating NSCs after stroke. During the early stage of stroke, astrocytes can restrict the diffusion of inflammatory factors by secreting various neurotrophic factors that protect nascent neurons. During the later stages, excessive proliferation and activation of astrocytes can mechanically hinder neural regeneration by forming an extensive network (33).

Additionally, TXC further augmented BDNF and TrκB levels, which likely increased NSC proliferation and differentiation in the SVZ, although the exact mechanism is still unclear. Studies have shown that BDNF and its receptor, TrκB, are upregulated following cerebral ischemia and epilepsy (34,35). Brain transplantation of human NSCs overexpressing BDNF provided differentiation and survival abilities to the grafted human NSCs and renewed angiogenesis in the host brain and functional recovery of ICH animals (36). The simultaneous increase in astrocyte proliferation and BDNF/TrκB expression after ICH suggests that activated glial cells secrete neurotrophic factors, which may drive pathological changes associated with ICH and promote neuronal survival. However, BDNF protein is mainly expressed in activated microglia around hematoma. In contrast, BDNF mRNA is mainly expressed in neurons and partially expressed in activated microglia around hematoma (37), which may be closely associated with the secretion of various neurotrophic factors by activated glial cells (38).

Taken together, the present findings demonstrated that TXC could significantly improve neurological deficits, absorption of brain edema, BBB integrity and NSC proliferation and differentiation into glial cells by upregulating the neuroprotective factors, BDNF and TrκB. This study showed that TXC might be an effective treatment method using an ICH rat model and provided a novel perspective on the treatment and prevention of ICH in patients. However, further clinical studies will be necessary to confirm these results.

## Data Availability

The datasets used and/or analyzed during the current study are available from the corresponding author on reasonable request.
